# A case of posterior urethral valve diagnosed by ultrasound in antenatal and pathology: A case report

**DOI:** 10.1097/MD.0000000000040961

**Published:** 2024-12-20

**Authors:** Jinmei Gao, Yunping Guan, Junling Kang, Wei Zhang

**Affiliations:** a Department of Ultrasound, Shenyang Maternity and Child Health Hospital, Shenyang, Liaoning Province, China; b Department of Pathology, Shenyang Maternity and Child Health Hospital, Shenyang, Liaoning Province, China; c Department of Pathology, Fukuang General Hospital of Liaoning Health Industry Group, Fushun, Liaoning Province, China.

**Keywords:** pathology, posterior urethral valve, prenatal diagnosis, ultrasound

## Abstract

**Rationale:**

Posterior urethral valve is a rare disease, prenatal diagnosis and prognosis evaluation are particularly important.

**Patient concerns:**

A 25-year-old pregnant woman was found enhanced parenchymal echo in both kidneys, subcapsule urinary cyst formation in both kidneys, bladder enlargement of the fetus during prenatal ultrasonography at 25 W + 4 of gestation. It was accompanied by fetal pericardial effusion and oligohydramnios.

**Diagnoses:**

Fetal posterior urethral valve.

**Interventions:**

After detailed prenatal evaluation, labor was induced and fetal autopsy was performed.

**Outcomes:**

Fetal autopsy pathology confirmed the prenatal judgment.

**Lessons:**

Prenatal ultrasonography can diagnose posterior urethral valve through typical image manifestations and can make a certain judgment for prognosis evaluation.

## 1. Introduction

Although the posterior urethral valve is the most common cause of lower urinary tract obstruction, it is also a rare disease that occurs only in males. Prenatal diagnosis is particularly important because it can lead to dysplasia and dysfunction of the urinary and other systems. Based on the typical case, this paper mainly emphasized the main prenatal ultrasonography of the prenatal and posterior urethral valve, and analyzed some pathologic characteristic on the gross pathology and microscopic pathology after induction of labor comparing with the prenatal ultrasonic performance, and further analyzed the factors that affect the prognosis and the thinking for differential diagnosis when the bladder enlargement was found before delivery. It is hoped that this case can provide some help for prenatal diagnosis and prognosis assessment of posterior urethral valve.

## 2. Case presentation

**Figure 1.**
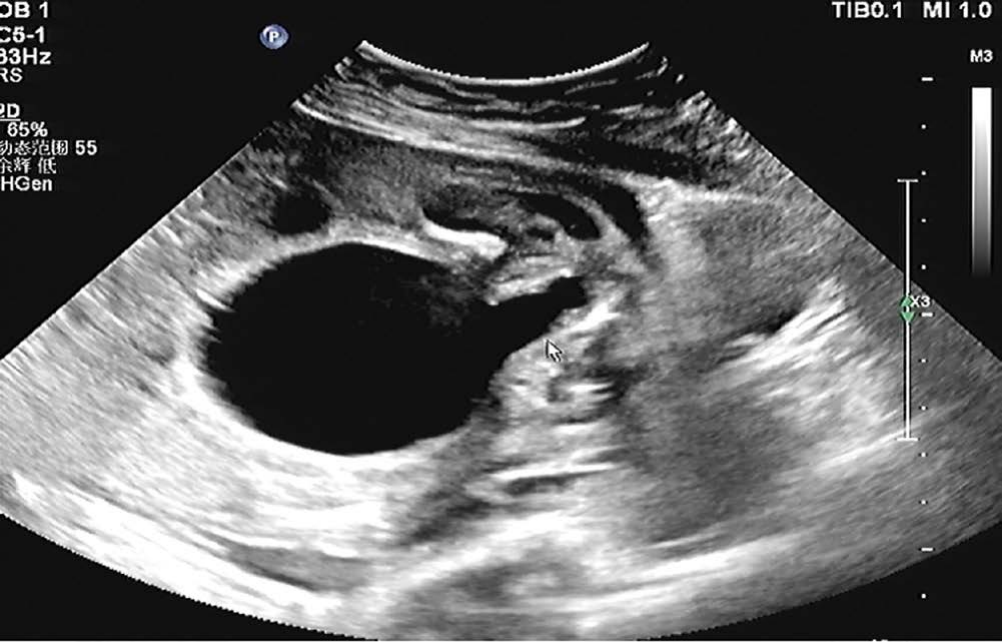
The bladder is enlarged, the bladder wall is thickened, and the proximal urethra is dilated, showing a “keyhole” sign. BL = bladder, PU = posterior urethra.

**Figure 2.**
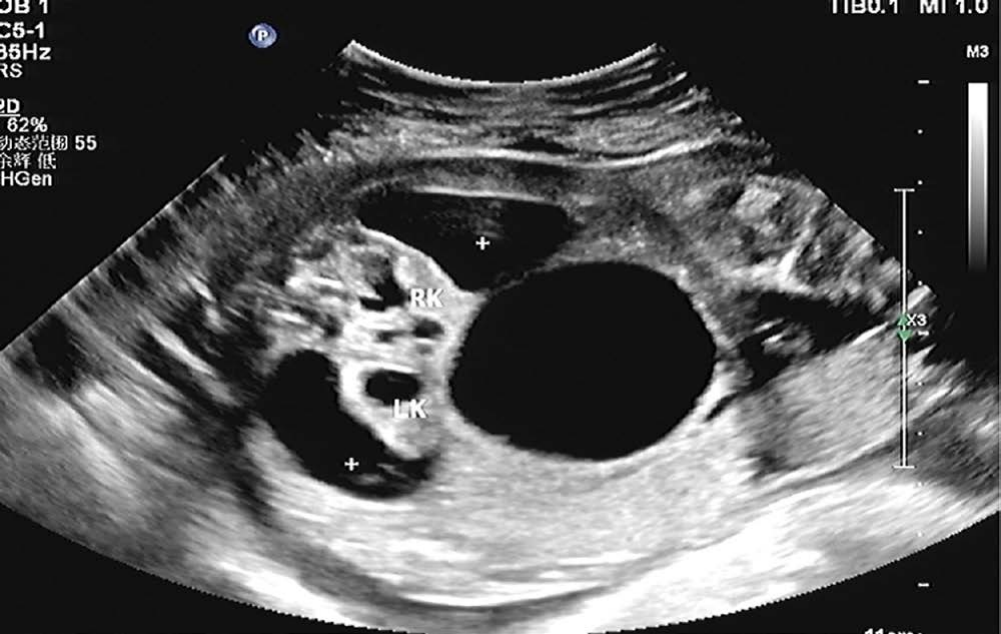
Enhanced echo in both kidneys, unclear boundary of renal cortex and renal medulla (LK, RK marks), perirenal urinary cyst formation (+ sign). LK = left kidney, RK = right kidney.

**Figure 3.**
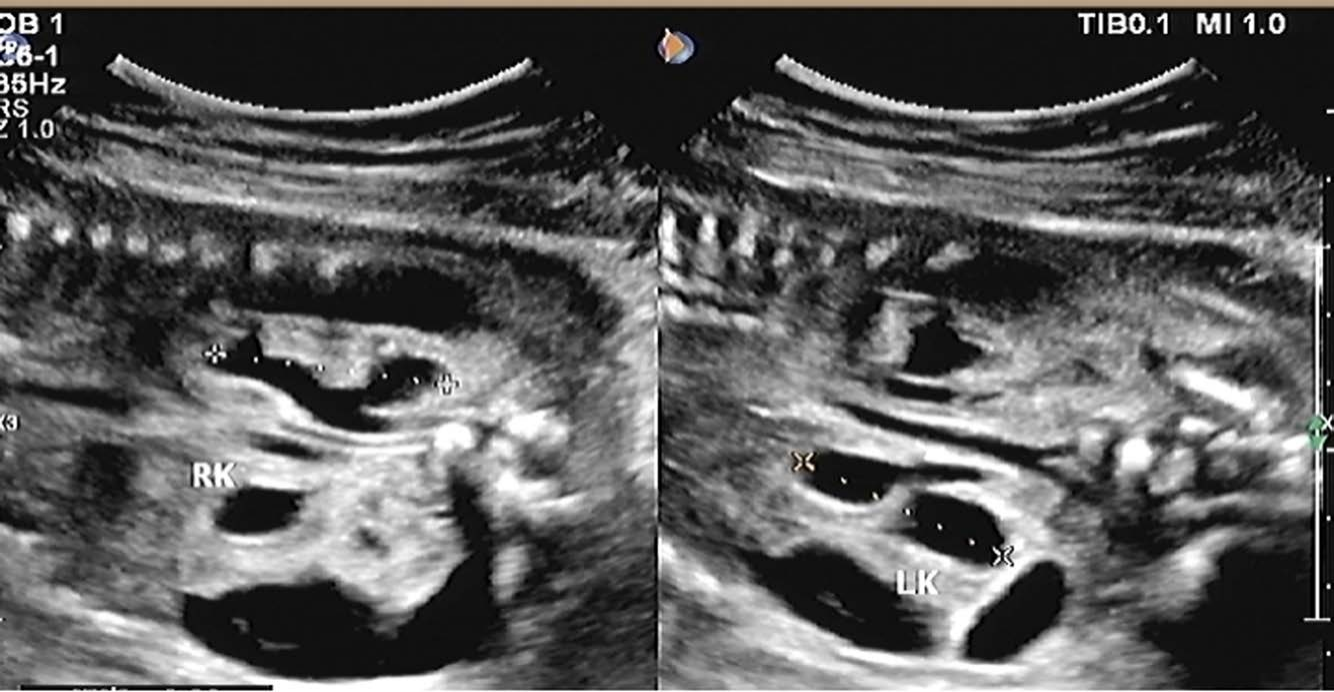
Double hydronephrosis. LK = left kidney, RK = right kidney.

A 25-year-old pregnant woman at 25 W + 4, G3P1. The patient had irregular menstruation. In early pregnancy, there was no history of vaginal bleeding or fetal preservation, and no obvious nausea or vomiting. There had no history of adverse drug exposure during the first trimester of pregnancy. She underwent regular prenatal checkups during pregnancy, and the risk of Down’s screening was low. The patient’s blood pressure was normal during pregnancy. She has not performed OGTT screening. The pregnant woman had a smoking history for 7 years with about 20 cigarettes a day before pregnancy, but she had no history of smoking during pregnancy. She denied a history of alcohol consumption or exposure to poison and dust. She had no history of contact with radioactive substances and denied a history of prostitution. The biological ultrasound measurements of the fetus were as follows: BPD: 5.6 cm, HC: 23.3 cm, AC: 23.5 cm, FL: 4.7 cm, HL: 4.3 cm, fetal weight, 947 ± 139 g. Ultrasonic scanning of the fetal structure showed that the cardiothoracic ratio was enlarged, and the pericardium showed a liquid dark area of approximately 0.31 cm deep. The left kidney was about 3.9 × 2.2 cm in size, which with enhanced parenchymal echo and unclear boundary of renal cortex and renal medulla. The separation range of the renal collecting system was approximately 2.9 × 1.0 cm, and the echo-free zone around the kidney was approximately 4.1 × 2.3 cm. The size of the right kidney was about 4.2 × 2.1 cm, the parenchymal echo was enhanced, the boundary of renal cortex and renal medulla was unclear, the separation range of the renal collecting system was about 3.0 × 1.0 cm, and the echo-free area was about 4.4 × 2.1 cm around the kidney. Dilation of the upper ureter was observed on both sides, with a width of 0.45 cm on the left and 0.40 cm on the right. The bladder was significantly enlarged, approximately 5.7 × 4.5 cm in size. The bladder wall was thickened by approximately 3.8 mm (>3 mm), and the proximal urethra was dilated (Figs. 1–3). The amniotic fluid depth was 2.0 cm and the amniotic fluid index was 3.1. There were no obvious abnormalities in the fetal structure. The ultrasound diagnoses were as follows: breech position of a single fetus in the second trimester; enhanced parenchymal echo of both kidneys, formation of subcapsular urinary cysts in both kidneys, enlarged bladder considering the posterior urethral valve; fetal pericardial effusion; oligohydramnios. No abnormality was found in other examination during pregnancy.

## 3. Interventions and pathology

After a detailed prenatal consultation, the pregnant woman chose to induce labor and fetal autopsy was performed. The operation was successful and the patient was discharged from hospital after 3 days.

**Figure 4.**
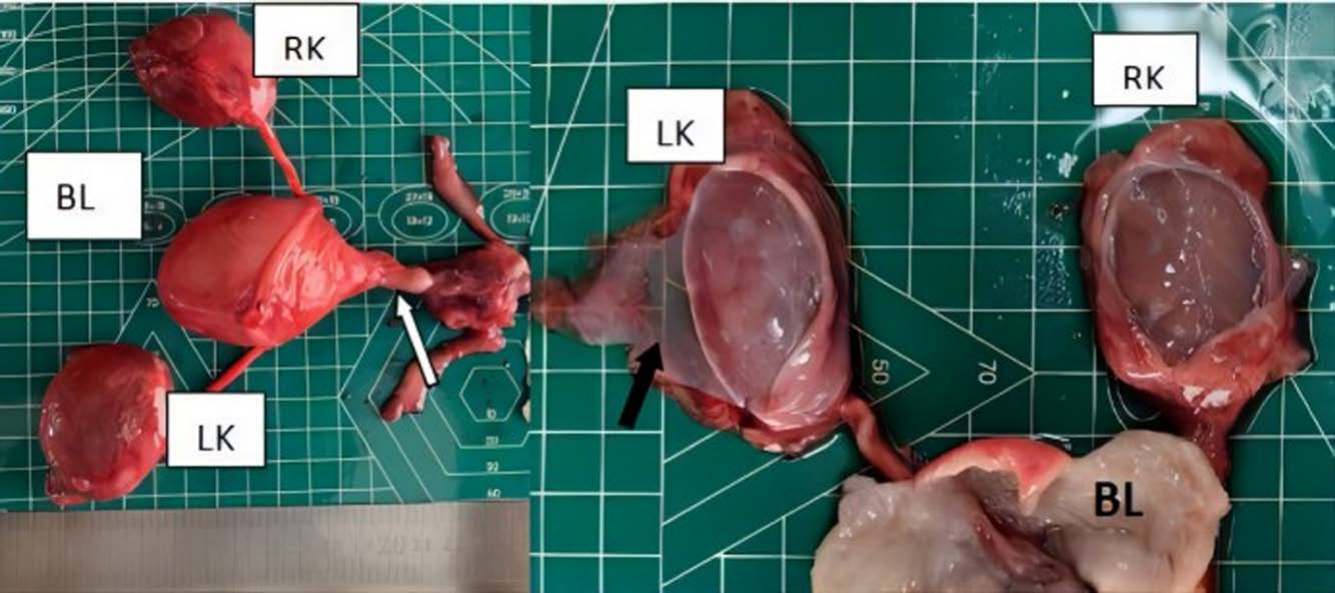
The bladder is enlarged, the posterior urethra is dilated at the proximal urethra (indicated by the white thick arrow), and urinary cysts around both kidneys are visible after incision, thin-walled renal fibroscan structure can be seen (indicated by the black thick arrow). BL = bladder, LK = left kidney, RK = right kidney.

**Figure 5.**
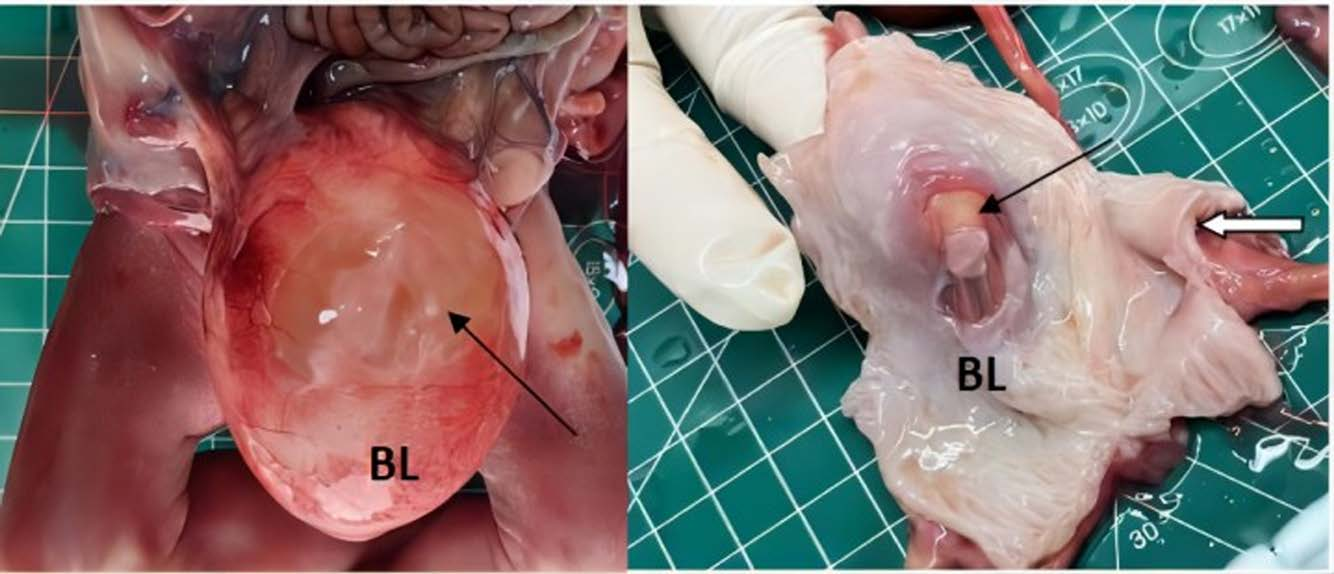
Local muscle layer in the posterior wall of the bladder (where the black arrow points) was absence. The bladder wall is thickened (where the white thick arrow points). BL = bladder.

**Figure 6.**
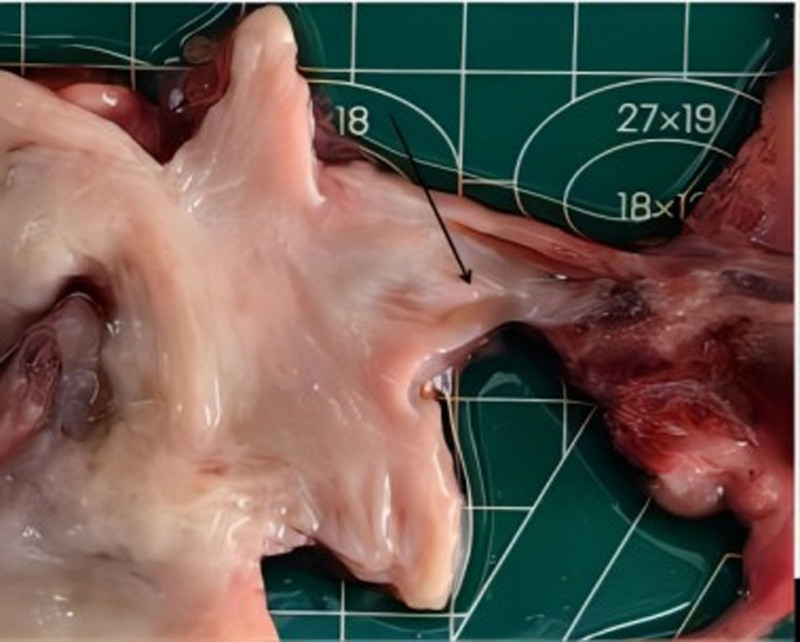
A longitudinal fold was showed in the enlarged posterior urethra (where the black arrow points).

**Figure 7.**
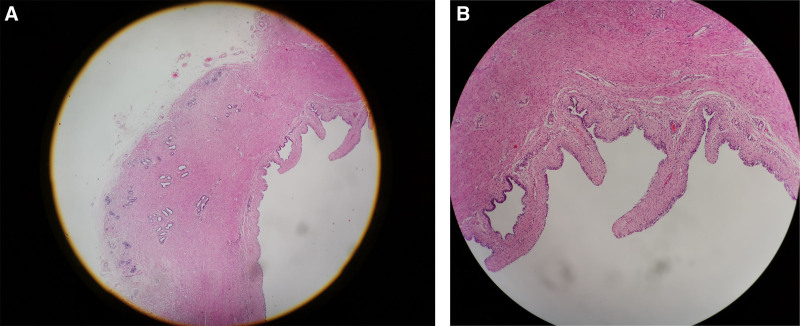
In the prostatic urethra (extended posterior urethra with longitudinal fold), local mucous membranes showed papillary protrusions, the surface is covered with laminated epithelial cells, and the mesenchyme is smooth muscle tissue and vascular tissue. (A) HEX40 and (B) HEX100.

**Figure 8.**
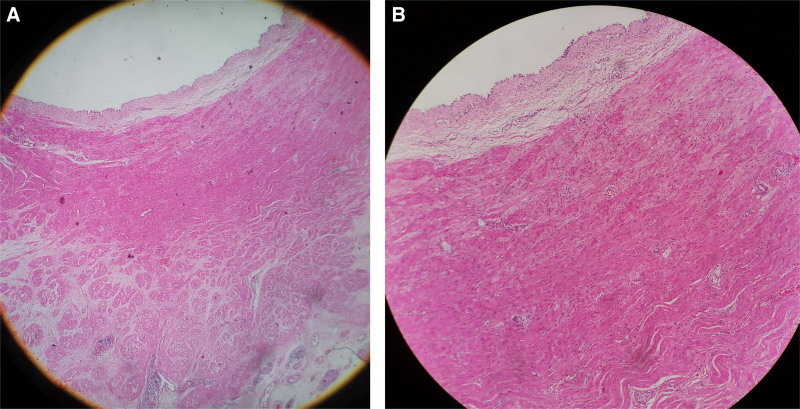
Bladder: The surface of the mucous membrane of the bladder is covered by a single layer of epithelial cells, the local epithelium is shed, and the muscular layer of the bladder is hyperplasia. (A) HEX40 and (B) HEX100.

**Figure 9.**
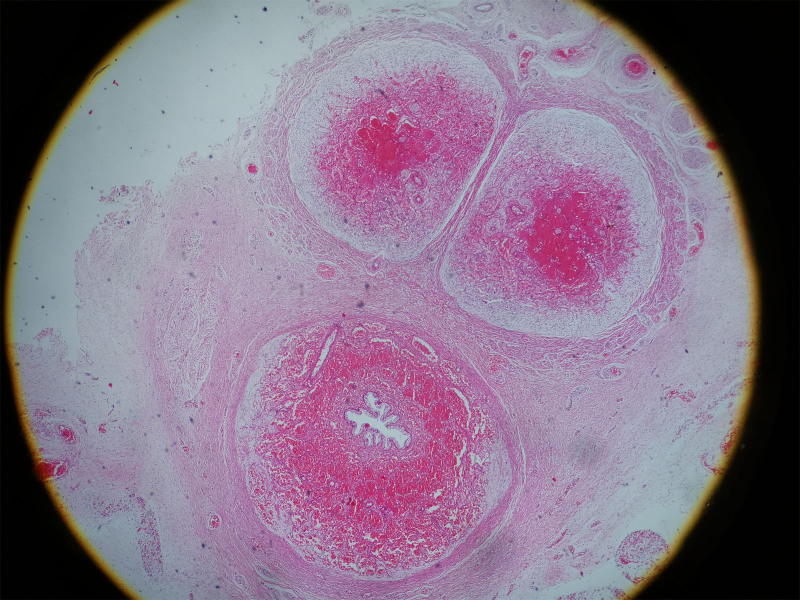
Urethra in the cavernous part of the penis. The histiocytic structure of urethra is normal, and normal urethra lumen can be seen (HEX40).

**Figure 10.**
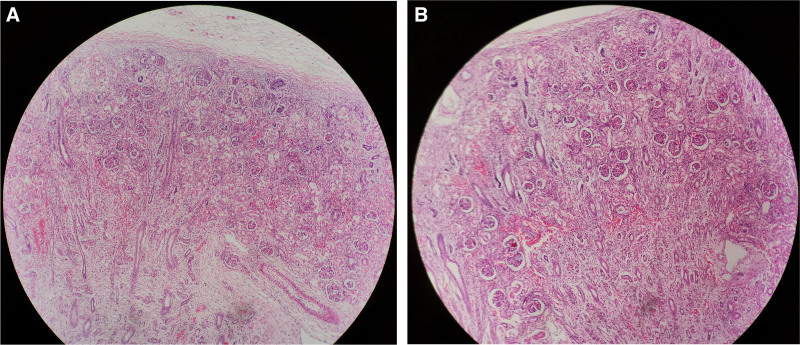
Kidney, the renal parenchyma is a normally differentiated renal histiocytic component. (A) HEX40 and (B) HEX100.

The gross pathology (Figs. 4–6) after induction of labor showed that the bladder was significantly enlarged, the bladder wall was thickened, some muscle tissue was missing in the posterior wall of the bladder, the proximal urethra was dilated, longitudinal raised plication-like structures were visible after sectioning, bilateral ureters were thickened, and light yellow urine accumulated in the renal fibrous sac. Microscopic pathology: urethra in the prostatic part (longitudinal fold of enlarged posterior urethra, Fig. 7): local mucous membranes showed papillary protrusions covered with multilayer epithelial cells, the mesenchyme was smooth muscle tissue and vascular tissue, and prostate gland components were observed around the enlarged urethra. Bladder (Fig. 8): The surface of the mucous membrane of the bladder was covered with a single layer of epithelial cells, the local epithelium was shed, and the muscular layer of the bladder was hyperplastic. Urethra in the cavernous part of the penis (Fig. 9): The histiocytic structure of the urethra is normal, and a normal urethral lumen can be seen. Kidney (Fig. 10): The renal parenchyma is a normally differentiated renal histiocytic component, and there are no pathological manifestations of glomerular dysplasia or tubular dysplasia under the microscope.

## 4. Discussion

The posterior urethral valve (PUV) is a congenital obstruction of the urethra. As this disease occurs in the first trimester, the bladder and upper urinary tract are exposed to high pressure throughout development, leading to profound changes in bladder function and kidney damage. PUV is the most common cause of lower urinary tract obstruction in male infants, and urinary tract disorders with various clinical features after birth due to urethral obstruction are the most common cause of chronic kidney failure in boys. While the incidence has remained stable, neonatal mortality from this disease has improved owing to early diagnosis and neonatal intensive care, partly due to the widespread use of prenatal ultrasound assessment. Prenatal ultrasonography usually shows bilateral hydronephrosis with bladder dilation and thickening, prostatic urethral dilation, and bladder neck hypertrophy. More severe fetuses may also develop urinary ascites or oligoamniotic fluid and echo or cystic kidneys. A postnatal voiding cystourethrogram can confirm the diagnosis by revealing a blocked urethral valve.

The posterior urethral valve is characterized by fetal bladder dilation and bladder wall hypertrophy due to increased pressure in the bladder caused by obstruction. The bladder fills the lower abdominal cavity, ureter is usually dilated, and degree of hydronephrosis varies. The valvular structure within the posterior urethra cannot be directly observed by prenatal ultrasound; however, the proximal end of the posterior urethra is significantly enlarged, and the bladder wall is thickened, forming a typical “keyhole” sign. In addition to typical bladder enlargement, formation of typical “keyhole” sign, and hydronephrosis of both kidneys, this case also presented with perirenal urinary cysts, which may have been caused by excessive pressure in the obstructed kidneys, rupture of the renal calyx, and urine leaking into the renal capsule, thus alleviating the intrarenal pressure and reducing the degree of hydronephrosis, which is consistent with the mild degree of hydronephrosis in this case. Gross and microscopic pathological findings further verified our prenatal ultrasound findings. The microscopic pathologic findings showed that there were multiple mucosal prominences in the expanded posterior urethra. We could not accurately identify small mucosal prominences with our naked eyes, and it was more difficult to find the valve fold in the prenatal period. The reduction of the bladder mucosa by compound epithelial cells, shedding of local epithelial cells, and proliferation of the bladder muscle layer are also closely related to the increase in bladder pressure and excessive expansion caused by obstruction. From the microscopic pathology of the cavernous urethra, we can clearly see the urethral lumen with a normal tissue structure, which further rules out urethral atresia. Although the kidney had ultrasonic manifestations of enhanced echo and unclear boundary of renal cortex and renal medulla, but, pathological renal cortical medullary differentiation was not found under the microscope, and there was not any manifestation of renal dysplasia or tubular dysplasia. Marie-Klaire Farrugia referred to in the article: early on in the obstructive process, bladder detrusor muscle architecture and function is maintained, but the effect on the obstructed kidneys is more acute, with evidence of hydronephrosis, cortical cysts with glomerular tufts and dilated medullary ducts.^[1]^ We analyzed that it may be attributed to the rupture of renal calices and urine extravasation into the renal fibrous sac, which relieved pressure in the kidney. It also further reduced kidney damage caused by stress, which is in line with some related research reports in the literature. The obstructed kidneys progressed from moderate hydronephrosis to renal dysplasia, glomerulus decreased by 20%, shunt hydronephrosis decreased, and the number of glomeruli increased; therefore, the investigators concluded that urinary transfer before the end of nephrogenesis could restore this process.^[2]^

Prenatal determinant of long-term renal outcome in patients with posterior urethral valve, as a single test, oligohydramnios had the best predictive accuracy with a sensitivity of 0.60 and a specificity of 0.74. When oligohydramnios, cortical cysts, and echogenic kidneys were combined, the specificity was 0.91, with a high positive likelihood ratio of 5.4. This indicates that if the scan is positive for all 3 characteristics, there is a high likelihood that the patient will develop end-stage renal disease. Univariate regression analysis showed that end-stage renal disease and chronic kidney disease were associated with decreased fetal age, oligohydramnios, renal cysts, oligohydramnios, cortical cysts, and renal echo combinations.^[3]^

In prenatal staging studies of congenital lower urinary tract obstruction, gestational age at the onset of oligohydramnios showed excellent accuracy in predicting the risk of perinatal death with the best cutoff of 26 weeks of gestation. Infants with normal amniotic fluid volume at 26 weeks of gestation had a lower risk of poor prognosis, defined as mild lower urinary tract obstruction; those with BV < 5.4 cm^3^ and normal amniotic fluid volume at 20 weeks of gestation were moderate; and those with BV ≥ 5.4 cm^3^ or abnormal amniotic fluid volume were severe before 20 weeks of gestation. The risk of perinatal death increased significantly from 9% to 26% to 55% depending on the severity from mild to moderate to severe. Similarly, the risk of severe renal impairment increases from 11% to 31% to 44% for mild, moderate, and severe lower urinary tract obstruction, respectively.^[4]^ When children are diagnosed early, urethral obstruction can be quickly alleviated by catheter insertion and eventual surgery, and metabolic disorders can be normalized in time to avoid preventable infant death.

Thus, the gestational week in this case was <26 weeks, along with oligohyniotic fluid, enhanced renal echo, and perirenal urinary cyst formation, all of which predicted a poor prognosis.

Distinguish from other diseases that cause bladder enlargement is need in antenatal:

1. Urethral atresia manifests as complete membranous obstruction of the urethra below the colliculi verticillaris, accompanied by distal hypoplasia of the urethra.^[1]^ The typical ultrasonographic manifestations of urethral atresia are fetal bladder enlargement, abnormal morphology, and plication-like structures protruding into the bladder are often visible, and the absence of amniotic fluid may be accompanied by hydronephrosis. Urinary peritoneal effusion caused by bladder rupture and urine entering the abdominal cavity after bladder rupture are rare complications. In this case, postmortem pathology showed that the urethral structure of the distal dilated urethra was normal, which ruled out urethral atresia.
2. Vesicoureteral reflux: Usually, it shows bladder enlargement and hydronephrosis, and the degree of hydronephrosis changes during dynamic observation without posterior urethral dilation on ultrasonography. The thickness of the bladder wall and amniotic fluid volume were mostly normal, and urinary ascites and perirenal urinoma did not occur. Prenatal ultrasound can reasonably and accurately distinguish male fetuses diagnosed with PUV and vesicoureteral reflux after delivery.^[5,6]^
3. Prune belly syndrome: Prune belly syndrome is a rare congenital acquired disorder, also known as Eagle-Barrett syndrome or Triad syndrome. It is characterized by defects in abdominal muscle tissue, cryptorchidism, and urinary tract abnormalities. In addition to the characteristic triad, approximately 75% of children with prune belly syndrome have abnormalities involving other systems, including respiratory, cardiac, gastrointestinal, and musculoskeletal defects.^[7]^ Prenatal diagnosis should be considered when the following ultrasonic abnormalities are present: oligohydramnios, urinary tract abnormalities (urethral dilation, giant bladder, giant ureter, and hydronephrosis), and loss of abdominal muscle tissue.^[8]^
4. Macrocystic-microcolon-delayed bowel peristalsis syndrome: It is a rare congenital disorder characterized by non-obstructing bladder dilation and microcolonic and reduced bowel peristalsis (dysperistalsis). It manifests as bowel obstruction and difficulty empting the bladder in newborns. Prenatally, it usually presents as an enlarged fetal bladder (macrobladder) and normal or increased amniotic fluid. Bowel dilation and excessive amniotic fluid were observed during the third trimester. The prevalence in female fetuses is 3 to 4 times higher than that in male fetuses, and the female sex is an important argument in support of the diagnosis.^[9]^

## 5. Conclusion

Prenatal ultrasonography can diagnose posterior urethral valve through typical image manifestations and can make a certain judgment for prognosis evaluation.

We hope to provide some case data for the study of posterior urethral valve through the prenatal ultrasound images and pathological manifestations after induction of labor of this case.

## Acknowledgments

We thank Dr Li Xinling for providing information about the patient and Dr Fan Rong for providing technical help on pathology.

## Author contributions

**Data curation:** Jinmei Gao.

**Formal analysis:** Jinmei Gao, Wei Zhang.

**Investigation:** Jinmei Gao.

**Methodology:** Junling Kang, Wei Zhang.

**Project administration:** Jinmei Gao.

**Supervision:** Yunping Guan.

**Validation:** Junling Kang.

**Writing – original draft:** Jinmei Gao.

**Writing – review & editing:** Jinmei Gao.
